# Novel avian calicivirus genome with type IV internal ribosomal entry site (IRES) in black-headed gull (Chroicocephalus ridibundus) in Hungary

**DOI:** 10.1007/s00705-026-06733-y

**Published:** 2026-09-02

**Authors:** Gábor Reuter, Benigna Balázs, Róbert Mátics, Ákos Boros, Péter Pankovics

**Affiliations:** 1 Department of Medical Microbiology, Medical School, University of Pécs, H-7624, Szigeti út 12, Pécs, Hungary; 2 Hungarian Nature Research Society, Ajka, Hungary; 3 Department of Behavioural Sciences, Medical School, University of Pécs, Pécs, Hungary

## Abstract

In this study, a taxonomically novel avian calicivirus detected and characterized by next generation sequencing, RT-PCR and Sanger sequencing methods in faecal specimen collected from black-headed gull (*Chroicocephalus ridibundus*) in Hungary. The complete genome length of the calicivirus strain gull/HA15097/HUN/2018 (PZ810127) is remarkably long, 8,845 nucleotides, which had type IV internal ribosomal entry site (IRES) at the 5’, and a stem-loop-II-like (s2m) sequence motif at the 3’ untranslated regions. The VP1 capsid protein had less than 26% aa identity to the members of the known calicivirus genera. Caliciviruses appear to be widespread not only in mammals including humans but also in various bird species.

Caliciviruses (family *Caliciviridae*) are non-enveloped, small round viruses with positive-sense, 6.4–8.5 kilobase long single-stranded RNA genome [1]. In general, the viral genome encodes two or three open reading frames (ORFs) including a large non-structural polyprotein (NSP), that is posttranslationally cleaved by the virus-encoded protease, followed by the structural proteins (VP1 major and VP2 minor capsid proteins). The caliciviral 5’ and 3’ genomic ends contains short, untranslated regions (UTRs) and translation initiation is mediated by viral 5’-terminal genome-linked protein (VPg) [2]. However, unexpectedly, some newly discovered avian caliciviruses [3–6] acquired a 500–900 nucleotide long 5’UTR with secondary RNA structure including type II, type IV (in ruddy turnstone calicivirus), or type V internal ribosomal entry sites (IRES) via horizontal gene transfer likely from picornaviruses on multiple occasions [7]. These structures allow the viral RNAs to utilize non-canonical 5’end-dependent mechanism of initiation.

The family *Caliciviridae* includes eleven official genera of which members of seven (*Lagovirus*,* Norovirus*,* Nebovirus*,* Recovirus*,* Sapovirus*,* Valovirus*, and *Vesivirus*) infect mammals, members of two genera (*Bavovirus* and *Nacovirus*) infect birds, and members of two genera (*Minovirus* and *Salovirus*) infect fish [1]. Furthermore, unclassified caliciviruses have been detected in amphibians and reptiles [1], highlighting the wide host range of caliciviruses among vertebrates.

Caliciviruses have been detected only from some of the ~ 11.000 known bird species. Until now, genetically highly different and even taxonomically partly or unclassified avian caliciviruses identified in domestic (e.g. chicken, turkey, duck, goose) and wild birds (e.g. pheasant, swan, cormorant, and other shorebirds, etc.) [3, 8–13]. It is likely that additional, yet unidentified caliciviruses may occur in further, different bird species. At present, the pathogenicity of avian caliciviruses in birds remains uncertain.

The black-headed gull (*Chroicocephalus ridibundus*) is a small-sized gull that breeds across the Palearctic including Europe and Asia. Most of the population is migratory, can be found in any wet habitat and winters coastal regions in Western Europe and North Africa but can occur as a vagrant in small numbers on every continent. It is a bold and opportunistic feeder. It eats insects, fish, seeds, worms, scraps, and carrion in towns, or invertebrates in ploughed fields (https://en.wikipedia.org/wiki/Black-headed_gull). In Urban areas, it also collects waste left behind by humans as a scavenger. Black-headed gulls are significant carriers of highly pathogenic avian influenza viruses [14, 15], but rotavirus and astrovirus have also been detected in them [16]. The black-headed gull’s lifestyle, its long-distance migration, and living proximity to humans make it an ideal reservoir and spreader of known and unknown microbes, including viral agents. Therefore, it would be important to explore the viruses of gulls.

This study reports the detection and complete genome characterization of a novel bird-origin calicivirus sequence identified in a black-headed gull.

A faecal specimen was collected from a black-headed gull (*Chroicocephalus ridibundus*) with bird ring number HA15097 in Sárrét, Fejér county, Hungary, in July 30, 2018. The sample was collected non-invasively by qualified ornithologists (R.M.) with valid permission (14/3859-9/2012, PE-KTF/97 − 13/2017), preserving the physical fitness of the birds. The ornithologist’s currently valid certificate (TMF-1180/22/2003) was issued by the National Inspectorate for Environment, Nature and Water. Next-generation sequencing (NGS) method was performed on this sample. A 200 µl PBS-diluted faecal sample (~ 40%V/V) was centrifuged at 6,000x g for 5 min and passed through 0.45 μm sterile PES membrane filters (Millipore/Merck, Germany). The filtrate was incubated with a mixture of DNases and RNases at 37 °C for 2 h to digest free/unprotected nucleic acids. Enriched viral nucleic acids (both RNA and DNA) were then extracted by the Quick RNA Kit (Zymo Research, Irvine, CA, USA) according to the manufacturer’s instructions. The NGS library was constructed by the xGen DNA EZ Library Prep Kit (IDT, Coralville, IA, USA). The library was sequenced on a NovaSeq X Plus (Illumina, San Diego, CA, USA) platform. Raw sequencing reads were first subjected to a comprehensive quality control and adapter trimming process using fastp (version 0.23.4) [17]. To ensure the removal of all technical artefacts, Sequence-Independent Single-Primer Amplification (SISPA) random primer sequences (5’-GCGGCCGCCACCAATTTAAAT-3’) and their reverse complements (5’-ATTTAAATTGGTGGCGGCCGC-3’) were manually specified for trimming, while the --detect_adapter_for_pe flag was employed to eliminate any remaining Illumina-specific adapter remnants. The resulting high-quality paired-end reads were then assembled into contigs using the SPAdes genome assembler (v4.0.0) [18] in metagenomic mode (--meta). During this process, k-mer sizes (ranging from 21 to 127) were dynamically and automatically selected based on the detected read length to optimize the assembly of potential viral sequences. To maximize the sensitivity of the analysis, all filtered reads were subsequently mapped back to the generated contigs using Bowtie2 (version 2.5.4) [19]. Reads that failed to align were extracted and pooled with the assembled contigs to create a comprehensive “master query” dataset, ensuring that viral signals not incorporated into the assembly were also preserved for downstream analysis. To maintain traceability and orientation during subsequent processing, sequences in the master query were assigned specific prefixes: assembled contigs were labelled with a “C_” prefix, while unmapped forward and reverse reads were designated with “R1_” and “R2_” prefixes, respectively. Final taxonomic classification and homology searches were performed using DIAMOND (v2.1.12.166) [20] in blastx mode. The master query was searched against the NCBI viral non-redundant (nr) protein database using the --very-sensitive setting. To ensure robust and reliable identification, a stringent E-value threshold of 1 × 10E-10 and a minimum amino acid identity of 40% were applied, with the search restricted to the top 5 target sequences per query. The resulting alignment data and taxonomic assignments were further processed, filtered, and visualized using a custom-developed Python-based application specifically designed for this pipeline.

The complete calicivirus genome sequence was verified after the total RNA isolation (TRI Reagent, MRC, Cincinnati, OH, USA) according to the manufacturer’s protocol (https://www.mrcgene.com/rna-isolation/tri-reagent). Overlapping internal regions of the viral genome were amplified by RT-PCR using a primer walking strategy with custom-designed virus-specific primers [21]. To sequence the 3’UTR, cDNA was synthesized using an anchored oligo(dT) adapter primer [22] and subsequently amplified with a virus-specific forward and an universal anchor reverse/adapter primers. The complete 5′UTR was determined via template-switching RT-PCR using the Template Switching RT Enzyme Mix (New England Biolabs, Ipswich, MA, USA) (https://www.neb.com/en/products/m0466-template-switching-rt-enzyme-mix according to the manufacturer’s 5′ RACE Protocol (https://www.neb.com/en/protocols/5-race-protocol-using-the-template-switching-rt-enzyme-mix). All purified PCR ampicons were subjected to bidirectional Sanger sequencing (3500 Genetic Analyzer, Applied Biosystems, Japan) using BigDye Terminator v3.1 Cycle Sequencing Kit.

The secondary RNA structure of the 5’UTR IRES was predicted by the Mfold web-based program [23] with visual correction taking into account the prototype type IV IRES structures [7].

A total of 10 additional faecal specimens were also collected from black-headed gull (*Chroicocephalus ridibundus*, N = 5) and Mediterranean gull (*Ichthyaetus melanocephalus*, N = 5) in Sárbogárd, Fejér county, Hungary in August 29, 2024 and tested for the novel calicivirus. Following RNA isolation, screening primers, F: 5‘-CCTTTGTACAATCYTGGAAYCA-3’ and R: 5‘-TGTTGCTGCCTATGTCTGCCAA-3’, designed for the VP1/ORF2 junction region of gull caliciviruses PX870883 and PZ810127 were used by RT-PCR and Sanger sequencing methods yielding a 1,001–1,031 nt long partial VP1 PCR product.

A total of 5,304,574 raw reads were generated by NGS method from the gull’s faecal specimen including 14,984 contigs. Among them, 365,490 reads had significant BLAST hits to viruses belonging to 2,681 virus species including eukaryotic virus families e.g. *Picornaviridae* (*N* = 47,933), *Caliciviridae* (*N* = 28,201), *Parvoviridae* (*N* = 15,716), *Sedoreoviridae* (*N* = 2,823), *Dicistroviridae* (*N* = 1,529), *Birnaviridae* (*N* = 1,393), and *Phenuiviridae* (*N* = 159). In this study, calicivirus sequences were selected for further analysis and complete genome characterization. From the 28,201 calicivirus reads, an 8,845 nucleotide-long contig was assembled which was representing the full-length calicivirus genome. The contig was confirmed by RT-PCR and Sanger sequencing methods. For verification of the genomeic sequence, a total of 1,066,530 calicivirus sequence reads (mean sequence coverage: 19,143 times) could be back-mapped using the complete, 8,845 nt long calicivirus study sequence, gull/HA15097/HUN/2018.

The gull calicivirus sequence strain gull/HA15097/HUN/2018 (PZ810127) had a 530 nt long 5’UTR, a 7,353 nt long ORF1 including the VP1 capsid-encoding region, a 612 nt long ORF2 (VP2) with a 13 nt long overlapping region with ORF1, and a 363 nt long 3’UTR excluding the poly(A)_n_ tail (Fig. 1). The nonstructural polyprotein including the VP1 major capsid protein contained the characteristic conserved calicivirus aa motifs (Fig. 1). Using GenBank BlastN search, only one nt sequence match was found with 8,828 nt-long calicivirus strain k141_266808 (PX870883) collected from a Southern black-backed gull from New Zealand in 2024 [24] with 82% nt identity (Query cover: 83%). The gull/HA15097/HUN/2018 ORF1 polyprotein is 2,450 aa (7,353 nt) long. Excluding the VP1 protein, the nonstructual protein had 97.6% (Query cover: 100%) and 51.3% (Query cover: 99%) aa identities to the corresponding polyprotein of calicivirus strain k141_266808 (PX870883) from Southern black-backed gull and calicivirus k141_105791 (PX870888) from South Island pied oystercatcher from New Zealand as the closest matches in GenBank, respectively. The gull/HA15097/HUN/2018 VP1 protein is 599 aa long and has 67.3% (Query cover: 100%) and 45.7% (Query cover: 97%) aa identities to the corresponding VP1 proteins of Southern black-backed gull calicivirus strain k141_266808 (PX870883) from New Zealand and Ruddy turnstone calicivirus A (MH453861) from Australia [3], as the closest matches, respectively. The gull/HA15097/HUN/2018 VP2 protein is 203 aa long and has 40.5% (Query cover: 55%) and 39.1% (Query cover: 89%) aa identities to the corresponding VP2 proteins of Ruddy turnstone calicivirus A (MH453861) and calicivirus (PV832500) from pigeon collected in China in 2024, as the closest matches, respectively.

**Fig. 1:**
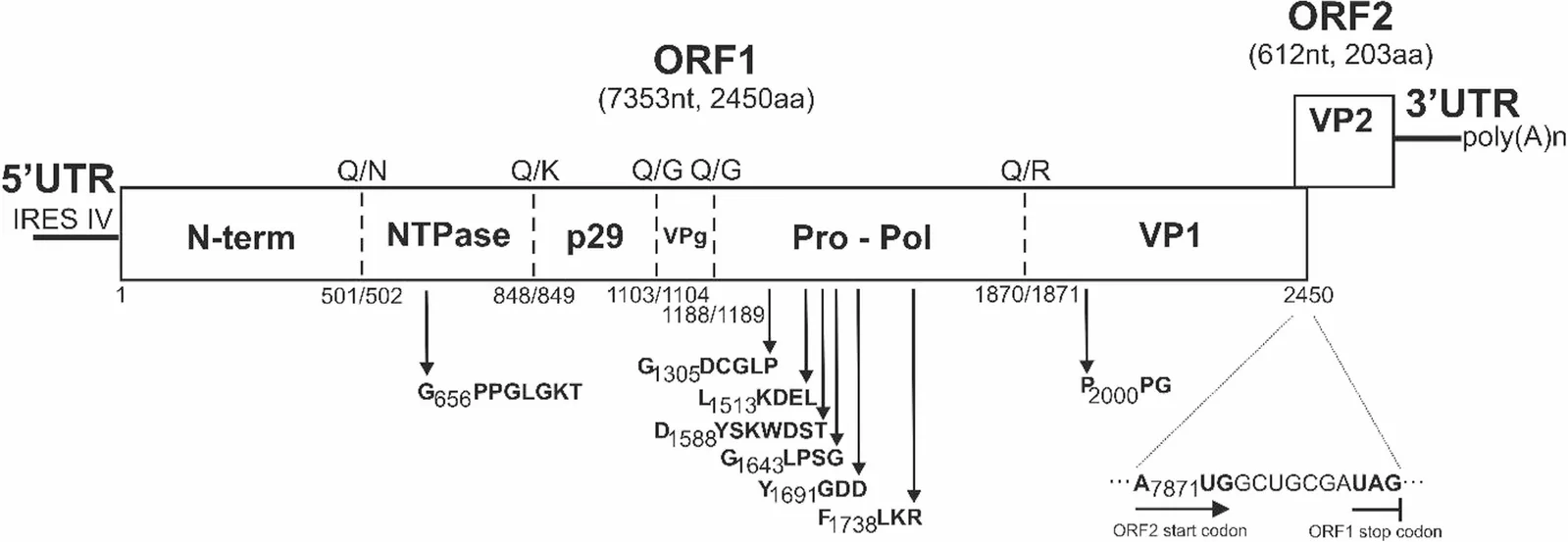
Schematic representation of untranslated (5’UTR and 3’UTR) and protein-coding regions (ORF1 and ORF2) in black-headed gull calicivirus (gull/HA15097/HUN/2018, PZ810127) genome. The potential cleavage sites are indicated above each part of the ORF1 encoding proteins with aa numbering below. The positions of the cleavage sites and the notations used in the figure follow those of Arhab et al. [7]. The nucleotide sequence is indicated at the ORF1/ORF2 junction. NTPase: helicase; VPg: viral protein genome-linked; Pro-Pol: protease-RNA-dependent RNA polymerase; VP: viral protein

The 5’UTR of gull/HA15097/HUN/2018 is 530 nt long. Using the GenBank BlastN, nucleotide sequence similarity (76–94%, Query cover: 10–98%) was found to 5’UTR IRES sequences of five unclassified avian caliciviruses (PX870883, PV832500, OR062614, PX870888, and ON815296) and seven picornaviruses (sapeloviruses from bird: PP908397; sapeloviruses from domestic pig: LC898902, LC508230; sapelovirus from rat: MW826551; anativiruses from bird: PV031098, AY563023; and megrivirus from bird: MK204390). Most of these 12 viruses are thought to be have type IV IRESs at the 5’UTR [7]. Based on this information a type IV IRES secondary RNA structure could be predicted in the black-headed gull calicivirus genome (PZ810127) from Hungary, as well as Southern black-backed gull calicivirus (PX870883) from New Zealand, too (Fig. 2).

**Fig. 2:**
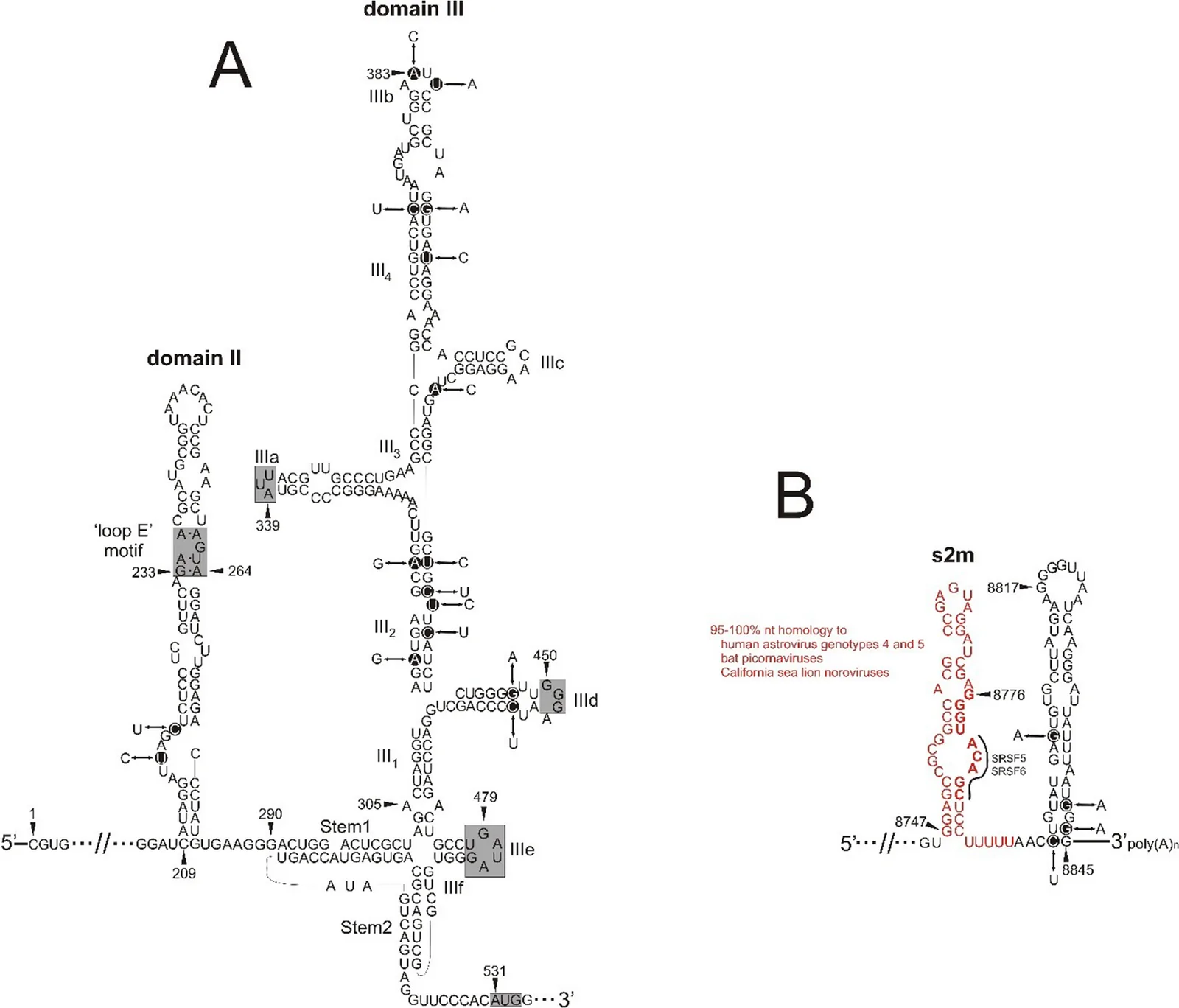
**A**) Predicted secondary RNA structure of the main domains (domain II and III) of type IV IRES in black-headed gull calicivirus 5’UTR (PZ810127). The individual helical segments of domain III are labelled: III_1_, III_2_, etc.; and the individual hairpins are labelled: IIIa, IIIb, etc. to maintain the continuity of the previously proposed IRES nomenclature [7, 29]. The positions of the conserved nucleotides and the nonstructural protein (ORF1) AUG start codon are highlighted by shaded boxes. The RNA secondary structure of black-headed gull calicivirus (PZ810127) from Hungary is supplemented with the (Southern black-backed) gull calicivirus 5’UTR IRES nucleotide sequence (PX870883) from New Zealand for comparison. The position of nucleotide differences is indicated by double arrows between the Hungarian gull calicivirus (dark circles) and the New Zealander gull calicivirus. All nucleotide mutations maintained the base pairing (and therefore the predicted secondary RNA structures) regardless of the Watson-Crick or wobble nature of base pairing. **B**) Predicted secondary RNA structure of the partial (~ 100nt long), terminal 3’ end of the 3’UTR of black-headed gull calicivirus (PZ810127) supplemented with the (Southern black-backed) gull calicivirus 3’UTR nucleotide sequence (PX870883) from New Zealand for comparison. Localization of homologus stem-loop-II motif (s2m) nucleotides (red color letters) to human astroviruses (e.g. AY188199, OP432301), bat picornaviruses (e.g. OR951243, OP963619) and California sea lion norovirus (MG572715) and conserved cell protein binding site (G_8776_GGUACAGC, red and **bold** letters) of cellular proteins (SRSF: serine/arginin-rich factors) [26] is indicated

The 3’UTR of gull/HA15097/HUN/2018 is 363 nt long. Using GenBank BlastN, 85% sequence similarity (Query cover: 97%) was found to 3’UTR sequences of Southern black-backed gull calicivirus (PX870883) from New Zealand. In addition, a very high (95–100%) nucleotide sequence homology was detected on a partial, 39–45 nucleotide-long 3’UTR genome sequences (corresponding to nt positions between 8,747 and 8,791 in black-headed gull calicivirus (PZ810127) especially to certain genotypes 4 and 5 human astroviruses, as well as bat picornaviruses and caliciviruses (California sea lion noroviruses) (Fig. 2). This region contains a highly conserved stem-loop-II-like (s2m) RNA structure and nucleotide motifs (e.g. GGGUACAGC) [25], that are thought to be binding sites of cellular proteins in astrovirus infections [26] (Fig. 2) and play a virus context-dependent role in viral replication [27].

A small-scale epidemiological investihation was conducted for the presence of the novel calicivirus among two gull species in Hungary. None of the 10 additionally available faecal specimens from gulls were RT-PCR positive for calicivirus using the screening primers.

Phylogenetic analysis based on the calicivirus non-structural protein (excluding the VP1) and VP1 capsid protein sequences shows that the study strain located on a bird-origin calicivirus lineage (Fig. 3). This avian lineage contains numerous caliciviruses that are significantly distinct genetically (and potentially antigenetically) from each other and currently not classified in any calicivirus genus or species. Based on the pairwise aa sequence comparison of the calicivirus VP1 proteins, gull/HA15097/HUN/2018 cannot be classified into any accepted calicivirus genus (Table 1).

**Fig. 3:**
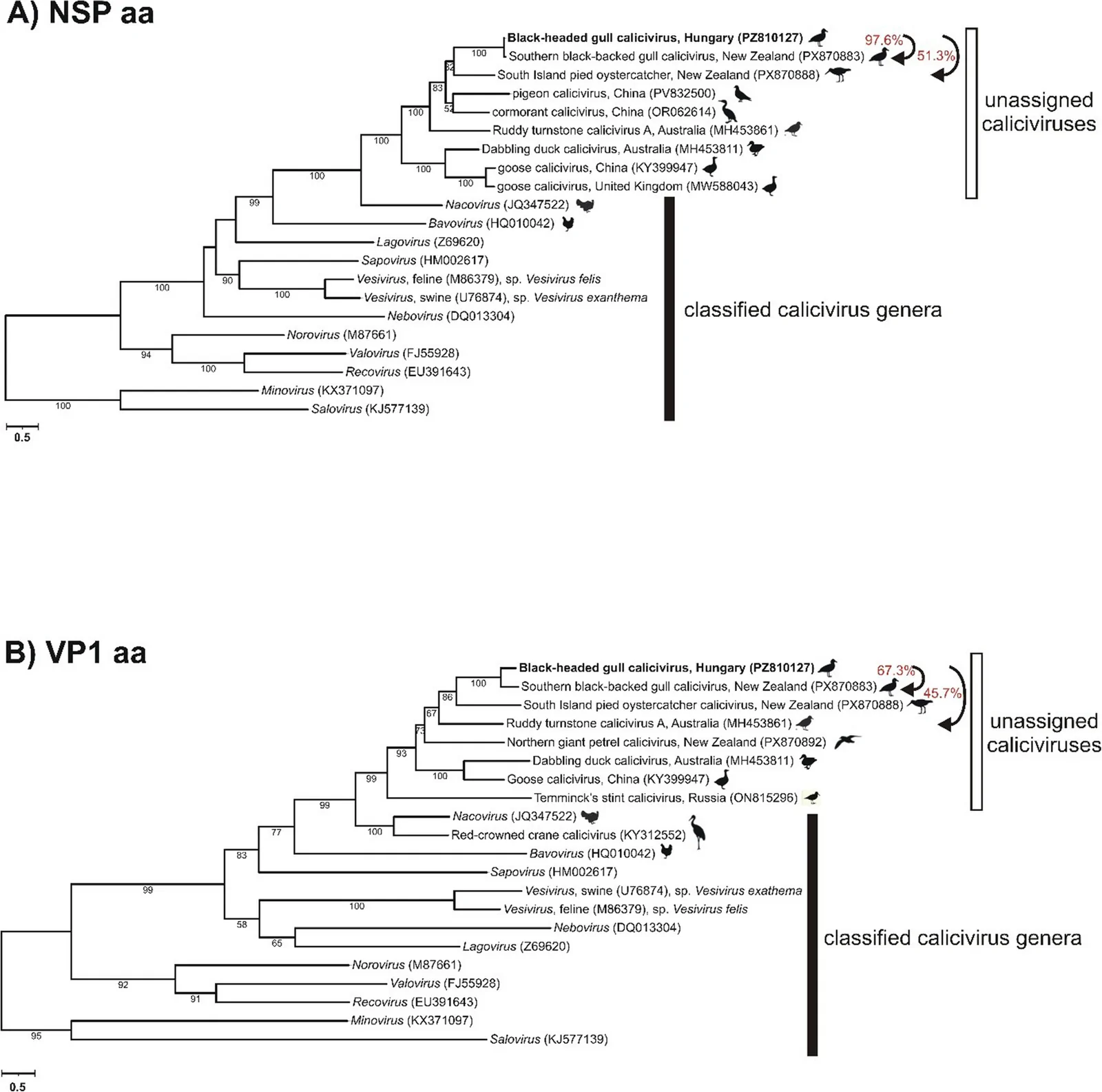
Maximum likelihood phylogenetic trees based on the calicivirus amino acid (aa) alignments of the **A**) complete non-structural polyprotein (NSP) excluding the VP1, and the **B**) VP1 capsid protein. The analyses included the study strain (shown in **bold**), representative prototype sequences from all known genera of the family *Caliciviridae* (marked with a black column) and the closest unassigned calicivirus sequences (marked with a white column) related to the study strain, as identified by BLASTp searches. Avian calicivirus sequences are marked with a silhouette characteristic of the given avian host species. Two species of the genus *Vesivirus* are labelled. The amino acid similarity of NSP and VP1 of the two closest caliciviruses to the study strain (in **bold**) is shown in percentage (red colour) in the figure (black arrows). The best-fit substitution models were selected according to the lowest Bayesian information criterion (BIC) using the IQ-TREE web server: Blosum62 + F+R5 for the NSP tree and LG + F+I+G4 for the VP1 tree. Maximum likelihood trees were generated in IQ-TREE with 1,000 ultrafast bootstrap replicates and visualised using iTOL and CorelDRAW Standard 2020 [30, 31]

**Table 1:** Pairwise amino acid sequence comparison based on the calicivirus VP1 protein in percentage (%) between gull/HA15097/HUN/2018 (PZ810127) and the corresponding protein of the prototypes strains in the calicivirus genera and the closest two available calicivirus strains in GenBank

Calicivirus genus (GenBank No. of the prototype strain)	Calicivirus animal hosts class	Percentage (%) of VP1 aa identity to gull/HA15097/HUN/2018 (PZ810127)
*Bavovirus* (HQ010042)	birds	13
*Lagovirus* (Z69620)	mammals	14
*Minovirus* (KX371097)	fish	8
*Nacovirus* (JQ347522)	birds	26
*Nebovirus* (DQ013304)	mammals	12
*Norovirus* (M87661)	mammals, human	11
*Recovirus* (EU391643)	mammals, human	10
*Salovirus* (KJ577139)	fish	10
*Sapovirus* (HM002617)	mammals, human	13
*Valovirus* (FJ55928)	mammals	10
*Vesivirus* (M86379)	mammals, human	10
The closest caliciviruses		
Southern black-backed gull calicivirus strain k141_266808 (PX870883)	bird	67
Ruddy turnstone calicivirus A (MH453861)	bird	45

In this study, we report the identification and genetic characterization of a novel avian calicivirus from a black-headed gull. Caliciviruses are known viruses in birds, but the diversity of caliciviruses and the number of bird species affected are certainly underestimated. So far, there is only one known case of calicivirus being detected (but not genetically analysed and peer-reviewed) in a gull from New Zealand in 2024 [24].

To our current knowledge, among caliciviruses, the study strain gull/HA15097/HUN/2018 has the longest calicivirus genome length (8,845nt) known to date, mostly due to the long 5’ and 3’UTR sequences. Caliciviruses generally have short non-coding regions at the ends of their genomes. An interesting exception is some avian caliciviruses, which have non-coding regions of several hundred nucleotides in length. These long 5’UTRs contain IRES structures homologous to picornavirus types I, IV, and V IRESes [7]. So far, only Ruddy turnstone calicivirus A (MH453861) was known to have a hepacivirus/pestivirus like type IV IRES [7] among the avian caliciviruses. Based on this study, two additional caliciviruses from gulls from New Zealand (PX870883) and Hungary (PZ810127), and presumably other avian calicivirus genomes (PV832500, OR062614, PX870888, and ON815296) possess this ~ 330 nt-long type IV IRES structures with conserved motifs in domains II and III. This perhaps means that type IV IRES is not uncommon in avian calicivirus genomes.

Interestingly, besides the picornavirus-like 5’UTR IRES, the calicivirus strain gull/HA15097/HUN/2018 (and its relatives PX870883 from New Zealand) contains an ~ 40–45 nt-long s2m-like highly conserved sequence motif at the 3’UTR part of the genome. This motif is present in some members of the viral families *Astroviridae*, *Caliciviridae*,* Coronaviridae*,* Picornaviridae*, and *Reoviridae* [27, 28] and originates from a wide range of host animals. Our results may confirms that recombination breakpoints occur not only between the ORF1 and ORF2 and between of the junction of the 5’UTR and ORF1 in caliciviruses but probably also between ORF2 and the 3’UTR, respectively, between different virus families.

According to the International Committee on Taxonomy of Viruses (ICTV), the genus demarcation criteria in the *Caliciviridae* family is the more than 60% amino acid sequence divergence of the VP1 [1]. Based on this, gull/HA15097/HUN/2018 cannot be classified in any of the currently recognized calicivirus genus. Together with its closest calicivirus relative (Southern black-backed gull calicivirus strain k141_266808, PX870883), they could be the founding members to a new calicivirus genus provisionally called “Birovirus” (*bir*d-*o*rigin calicivirus). It should be noted that the variability of VP2 sequences between caliciviruses is considerably high and the length of these proteins varies significantly [13]. The aa difference in the VP2 small capsid protein between the two gull caliciviruses (PZ810127 and PX870883) is also significant, more than 60%.

It should also be noted that in the tested gull faecal specimen, in addition to calicivirus, additional sequences from a large number of other, potentially novel viruses (e.g. at least three different picornaviruses, parvoviruses, phenuiviruses, etc.) and even bacteria (non-O1 or non-O139 serogroup *Vibrio cholerae*) could also be identified as co-infections. This also indicates that gulls encounter a wide range of microorganisms due to their lifestyle and potentially can carry them to distant geographical regions.

Genetically diverse caliciviruses appear to be widespread not only in mammals including humans but also in various bird species. Further studies are needed to describe the diversity, pathogenic role and biology of avian caliciviruses.

## Data Availability

No datasets were generated or analysed during the current study.
